# Cardiac tamponade: contrast reflux as an indicator of cardiac chamber equalization

**DOI:** 10.1186/1749-8090-7-48

**Published:** 2012-05-31

**Authors:** Foeke Jacob Harmen Nauta, Wernard Aat Antoine Borstlap, Michael Stella, Zain Khalpey

**Affiliations:** 1 The University of Arizona Medical Center - University Campus, 1501 North Campbell Avenue , Tucson, AZ, 85724, USA

**Keywords:** Cardiac tamponade, Hemopericardium, Blunt trauma, Contrast reflux, Cardiac hemodynamics

## Abstract

**Background:**

Traumatic hemopericardium remains a rare entity; it does however commonly cause cardiac tamponade which remains a major cause of death in traumatic blunt cardiac injury.

**Objectives:**

We present a case of blunt chest trauma complicated by cardiac tamponade causing cardiac chamber equalization revealed by reflux of contrast.

**Case report:**

A 29-year-old unidentified male suffered blunt chest trauma in a motor vehicle collision. Computed tomography (CT) demonstrated a periaortic hematoma and hemopericardium. Significant contrast reflux was seen in the inferior vena cava and hepatic veins suggesting a change in cardiac chamber pressures. After intensive treatment including cardiac massage this patient expired of cardiac arrest.

**Conclusion:**

Reflux of contrast on CT imaging can be an indicator of traumatic cardiac tamponade.

## Background

Traumatic cardiac tamponade remains a deadly, but rare, reason for presentation to the emergency department [1]. Its main cause in trauma patients is penetrating chest injury (80-90%) and it occurs in only 10% of blunt chest trauma injuries. Thoracic injuries account for approximately 25% of trauma related deaths and are a contributing factor in an additional 25% of deaths in the United States annually [1]. As it is typical for acute trauma, fast and accurate diagnostics are crucial. We present a case of blunt chest trauma including cardiac tamponade revealed by contrast reflux at CT.

## Case presentation

**Figure 1:**
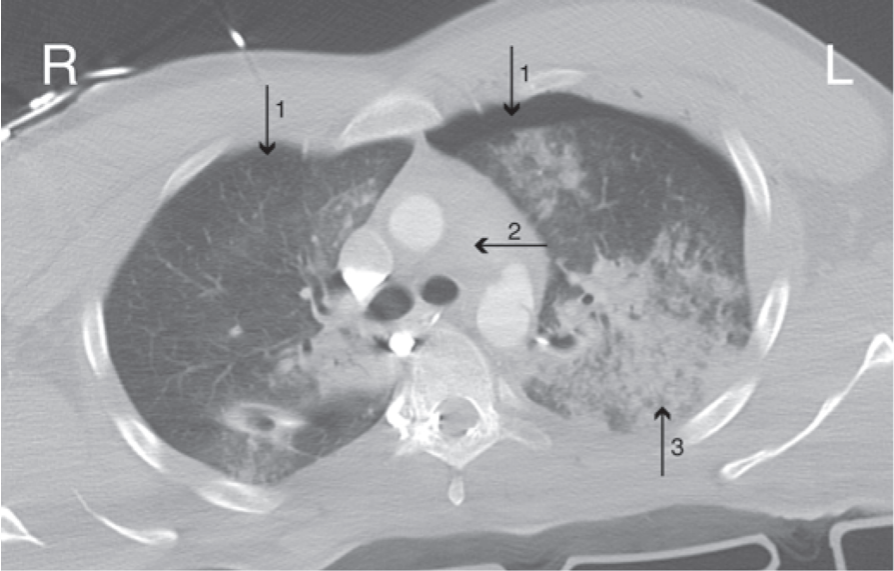
Axial contrast CT chest showed bilateral pneumothoraces (1), periaortic hematoma (2) and pulmonary contusion (3)

**Figure 2:**
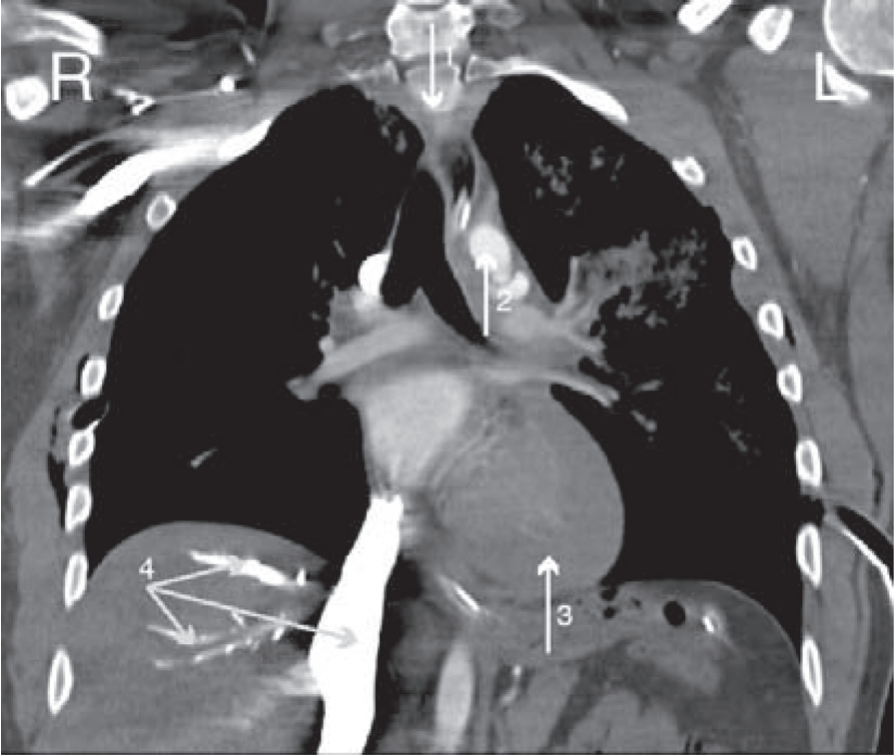
Coronal contrast CT chest showed mild right sided deviation of the trachea (1), pseudoaneurysm of the descending aorta (2), hemopericardium (3) and contrast reflux into the IVC and hepatic veins (4)

A 29-years-old unidentified male suffered multiple blunt trauma in a severe motor vehicle collision. On suspicion of intracranial injury due to left sided globe rupture, FAST exam was skipped and the patient was rushed to CT. After intravenous administration of contrast, axial CT (Figure 1) demonstrated bilateral pneumothoraces, periaortic hematoma and pulmonary contusion. Coronal view (Figure 2) showed mild right sided deviation of the trachea, pseudoaneurysm of the descending aorta and hemopericardium. Remarkable contrast reflux was seen into the inferior vena cava (IVC) and hepatic veins. This phenomenon was presumably caused by equalization of cardiac chamber pressures typical of tamponade. Even though emergent thoracotomy following blunt trauma carries an overall survival rate of only 1-2% [2], after the loss of vital signs, emergent thoracotomy was performed and 2.5 liters of pericardial blood was found originating from a right atrial tear. This case shows that the use of contrast in CT can reveal changes in cardiac chamber pressures possibly indicating traumatic cardiac tamponade.

### Discussion

Several studies have described the relationship between IVC contrast reflux and several morbidities (pulmonary hypertension, tricuspid regurgitation and right ventricular systolic dysfunction) [3-5]. Dusaj et al. [6] demonstrated the hemodynamic potential of Coronary CT angiography (CTCA). Quantification of IVC and SVC contrast characteristics during CTCA provides a quick, feasible and accurate method of estimating right atrial and ventricular response. Their findings correlated with echocardiographic estimations of right atrial and right ventricular pressures. Similarly, we have found that contrast reflux in the IVC, SVC and hepatic veins correlates with acute hemodynamic changes associated with cardiac tamponade.

## Conclusion

This case emphasizes the diagnostic potential of contrast enhanced CT of the chest to characterize acute cardiovascular physiology, particularly in blunt chest trauma.

## Consent

Unfortunately patient deceased and no medical consent was obtained.

## Abbreviations

CT: Computed tomography; FAST: Focused assessment with sonography for trauma; IVC: Inferior vena cava; CTCA: Coronary CT angiography; SVC: Superior vena cava.

## Competing interest

The author(s) declare that they have no competing interest.

## Authors’ contributions

FN and WB reviewed the literature, FN wrote the manuscript. ZK, WB and MS edited the text. MS supplied and described the images. All authors read and approved the final manuscript.

## Author’s information

FN and WB are clinical research trainees. ZK is the supervising chief resident cardiac surgery and MS is a radiologist.
